# The Role of Myeloid-Derived Suppressor Cells in Immune Ontogeny

**DOI:** 10.3389/fimmu.2014.00387

**Published:** 2014-08-13

**Authors:** Soren Gantt, Ana Gervassi, Heather Jaspan, Helen Horton

**Affiliations:** ^1^Child and Family Research Institute, University of British Columbia, Vancouver, BC, Canada; ^2^Seattle BioMed, Seattle, WA, USA; ^3^Division of Immunology, University of Cape Town, Cape Town, South Africa; ^4^Janssen ID&V Research and Development, Antwerp, Belgium

**Keywords:** myeloid-derived suppressor cells, immune ontogeny, feto-maternal tolerance, neonate

## Abstract

Myeloid-derived suppressor cells (MDSC) are a heterogeneous population of granulocytic or monocytic cells that suppress innate as well as adaptive immune responses. In healthy adults, immature myeloid cells differentiate into macrophages, dendritic cells, and granulocytes in the bone marrow and MDSC are rarely detected in peripheral blood. However, in certain pathologies, in particular malignancies and chronic infection, differentiation of these cells is altered resulting in accumulation of circulating suppressive myeloid cells. MDSC express suppressive factors such as arginase-1, reactive oxygen species, and inducible nitric oxide synthase, which have the ability to inhibit T cell proliferation and cytoxicity, induce the expansion of regulatory T cells, and block natural killer cell activation. It is increasingly recognized that MDSC alter the immune response to several cancers, and perhaps chronic viral infections, in clinically important ways. In this review, we outline the potential contribution of MDSC to the generation of feto-maternal tolerance and to the ineffective immune responses to many infections and vaccines observed in early post-natal life. Granulocytic MDSC are present in large numbers in pregnant women and in cord blood, and wane rapidly during infancy. Furthermore, cord blood MDSC suppress *in vitro* T cell and NK responses, suggesting that they may play a significant role in human immune ontogeny. However, there are currently no data that demonstrate *in vivo* effects of MDSC on feto-maternal tolerance or immune ontogeny. Studies are ongoing to evaluate the functional importance of MDSC, including their effects on control of infection and response to vaccination in infancy. Importantly, several pharmacologic interventions have the potential to reverse MDSC function. Understanding the role of MDSC in infant ontogeny and their mechanisms of action could lead to interventions that reduce mortality due to early-life infections.

## Introduction

Approximately, one million newborns die due to infection each year worldwide (1). It is increasingly apparent that the extreme vulnerability to infections of neonates and young children is related to substantial changes that the immune system undergoes during early life (2–6). In order to suppress allogeneic responses and pathologic inflammation due to antigenic differences between the fetus and its mother, the feto-maternal environment evolved to be immunosuppressive (7, 8), which likely influences post-natal immune responses. Furthermore, specific antigenic exposures or infectious risks that occur at different ages during childhood may have shaped the observed transition to more adult-type immune responses (9). It is clear that nearly all aspects of the neonatal innate and adaptive immune response differ from those of older children and adults; the underlying mechanisms for these differences are multifactorial, and as yet incompletely understood. This review will discuss the potential contribution of myeloid-derived suppressor cells (MDSC) to the ontogeny of the human immune system and early-life immunologic phenotype. MDSC are particularly intriguing because of their plasticity and the availability of agents to reverse their suppressive activity, which could prove valuable for accelerating the ability of young infants to mount protective immune responses to infection or vaccines. As such, we summarize the evidence and outline a proposed agenda of future research on MDSC with respect to newborn and infant immunity.

## MDSC Characterization and Phenotypes

Myeloid lineage progenitors generated in the bone marrow classically differentiate into macrophages, dendritic cells (DC), and granulocytes. The ontogeny of myeloid cells is discussed in detail elsewhere in this issue (De Kleer et al., submitted), and numerous excellent reviews of MDSC in other contexts have been published (10–15). MDSC are not a separate lineage of cells, but are rather a heterogeneous population of activated myeloid cells with suppressive functions. Although suppressive myeloid cells were described more than 30 years ago (16–18), the diverse phenotypes of MDSC and their biological roles have only recently begun to be characterized in detail. These cells are defined by having myeloid markers, potent immunosuppressive activity, and for monocytic MDSC, the ability to differentiate into mature macrophages and DC. In mice, there are two relatively distinct subsets of MDSC: monocytic MDSC (CD11b^+^ LY6G^−^ LY6C^high^) and granulocytic MDSC (CD11b^+^ LY6G^+^ LY6C^low^) (10, 13). Human MDSC are less easily categorized into monocytic vs. granulocytic because of the lack of a Ly-6G (Gr-1) gene homolog in humans. However, human MDSC have been defined as CD33^+^ CD11b^+^ HLA-DR^low/−^, with monocytic MDSC being CD14^±^ CD15^low/−^ and granulocytic MDSC being CD14^−^ CD15^+^ CD66b^+^, which appears consistent with hematologic morphology (10, 13). A population of promyelocytic MDSC in bone marrow and several cancers has also been defined by being CD33^+^ HLA-DR^−^ CD11b^low/−^ CD16^−^ (15, 19). MDSC populations appear to be predominantly of the granulocytic type in the setting of cancer, as well as in early infancy, as discussed below. In HIV and other chronic diseases, the relative importance of monocytic and granulocytic MDSC is unclear (11, 20–22). These definitions and classifications are somewhat controversial, however, given the heterogeneity of MDSC populations and the variability in markers used by different groups, and the fact that there may be overlap between MDSC phenotypes (23, 24). Furthermore, it is not entirely clear whether or how granulocytic MDSC differ from other subsets of activated mature neutrophils with suppressive activity (25).

## Suppressive Mechanisms

The hallmark of MDSC is their ability to suppress T cell and NK cell responses. MDSC have been shown to suppress immune responses through a variety of direct mechanisms, including arginase-1, inducible nitric oxide synthase (iNOS), and production of reactive oxygen species (ROS). Both arginase-1 and iNOS metabolize arginine. In humans, arginase-1 is primarily expressed in granulocytic MDSC, whereas in mice, arginase-1 is expressed by both monocytic and granulocytic MDSC (26). However, in both species, the downstream effects of arginase-1 appear the same. l-arginine is catabolized by arginase-1 to urea and l-ornithine. In humans, arginine starvation inhibits T cell proliferation through decreasing CD3ζ-chain expression (27) and preventing the expression of cell-cycle regulators cyclinD3 and cdk4 (28). Taheri et al. first demonstrated that Jurkat T cells cultured in medium with levels of arginine <50 μM (normal levels of arginine in the serum range between 50 and 150 μM) resulted in the loss of CD3ζ expression (29). Down-regulation of TCR ζ-chain is known to be critical for normal T cell function, including proliferation and IFNγ production (30).

MDSC-derived iNOS converts l-arginine to citrulline and NO, which suppresses T cell function through inhibition of Jak/STAT signaling, reducing MHC class II expression and inducing T cell apoptosis (31–34). ROS and NO produced by MDSC also result in nitration of the T cell receptor, interfering with recognition of peptide antigens presented by MHC (35). Because cysteine provided by antigen-presenting cells (APC) is required for T cell activation, MDSC also inhibit T cell responses by depleting the pool of cysteine available to APC (36). MDSC can also inhibit T cell responses in a contact-dependent manner, such as through membrane-bound TGF-β (37). Tumor models have also demonstrated direct suppression of NK cell cytotoxicity, NKG2D expression, and IFN-γ production by MDSC in a contact-dependent manner (37, 38). Arginase-1 has also been shown to inhibit NK cell proliferation and secretion of IFN-γ (39).

Other mechanisms of MDSC suppression in various models and disease states include their expression of program death ligand 1 (PD-L1), CD80/86 (the ligand for CTLA4), and Galectin-9 (the ligand for Tim-3), as well as production of heme oxygenase-1, IL-6, and IL-10 (40–43). In addition to acting on T cells directly, as tolerogenic APCs, MDSC also suppress T cell responses indirectly, through other suppressive cell populations. Regulatory T cells (Tregs) are recruited and expanded by MDSC production of TGF-β and IL-10 and through CD40–CD40L interactions (44–46). In addition, IL-10 production by MDSC may also influence T cell function via macrophages, which produce less IL-12 and predispose to Th2-type responses (47).

## Expansion and Role in Pathologic Conditions

In healthy adults, immature myeloid cells that are generated in the bone marrow differentiate into mature, functional macrophages, DC, and granulocytes. However, in certain pathologic conditions, in particular cancer, there is accumulation and activation of MDSC that can potently suppress T cell and NK cell function (10, 12, 13). In addition to their importance in cancer pathogenesis, studies demonstrate a role for MDSC suppressive function during chronic infections/inflammation, sepsis, transplant, and trauma (11, 22, 48–51). In particular, the suppressive effects of MDSC appear to impair control of chronic viral infections, both in mouse models using lymphocytic choriomeningitis virus and vesicular stomatitis virus, as well as in observational human studies of HIV and hepatitis C virus infections (11, 52). MDSC expand and become activated in response to a variety of factors, including inflammatory cytokines (IL-6, VEGF), other pro-inflammatory factors (COX2 and prostaglandin E_2_ (PGE_2_), GM-CSF, M-CSF, stem cell factor (SCF)-1 (10, 12, 13). In addition, MDSC may also be increased in elderly mice (53) and humans (54), which might be involved with the phenomena of immunosenescence and “inflammaging.”

Although induction of MDSC may be a normal physiologic response to inflammation, there is convincing evidence that they can be deleterious in malignant conditions (12–14). A number of chemotherapeutic interventions that target MDSC are being studied. Agents that decrease MDSC number include sunitinib, gemcitabine, and docetaxel; other drugs, such as COX-2 inhibitors and the phosphodiesterase-5 inhibitor sildenafil, appear to inhibit MDSC function (12, 14, 15). Perhaps most interestingly, use of ATRA (all-trans retinoic acid) (55, 56) or vitamin D3 (57, 58) has been shown to drive maturation of MDSC into fully functional stimulatory monocytes and DC.

## Role in Feto-Maternal Tolerance and Immune Ontogeny

There are clear differences in both innate and adaptive immune responses between neonates and older children or adults (2–6, 59). The fetus is antigenically different from its mother, and is thus analogous to a semi-allogeneic transplant, with the risk of immunologic rejection (7, 8). The fetal immune system is biased toward tolerogenic responses, as is that of the pregnant woman. Thus, the immune response during pregnancy appears to have evolved to prevent potentially damaging inflammation that may result in abortion or pre-term delivery. Furthermore, early post-natal tolerance might prevent damaging inflammation due to microbial colonization or environmental antigens in extrauterine life (60). Although the immunologic state of the fetus and newborn might be evolutionarily advantageous, evidence suggests that it predisposes to severe infections, especially those due to intracellular pathogens, and impairs responses to vaccinations in post-natal life (9, 59, 61). Innate immune cells – monocytes, DC, NK cells – respond differently in neonates compared to later in life, and this contributes to their vulnerability to infection (9, 62–66). In addition to altered innate immunity, adaptive responses are also qualitatively different in early life (61, 67–70). These differences likely in part reflect the innate neonatal response. However, neonatal T cells may also have intrinsic differences, with CD4^+^ T cell being biased toward Th2 responses and CD8^+^ effector T cells may be particularly short-lived (71, 72). As described elsewhere in this issue, multiple interrelated mechanisms likely contribute to the quality of the immune responses observed *in utero* and during early life. Included among them are suppressor cell populations, such as Tregs in the fetus (73), a novel immunosuppressive CD71^+^ erythroid cell type in newborns (60), and others (74). Preliminary findings suggest that MDSC may also contribute to feto-maternal tolerance and infant immune ontogeny.

Recent studies by Rieber et al. and by our own group have found that MDSC are present in high numbers in cord blood (75–77). As opposed to healthy adults, in whom <1% of PBMC are MDSC, CBMC of healthy neonates has a median of approximately 5% MDSC, comparable to frequencies observed in PBMC of cancer patients. Of note, there is substantial variation between individual neonates, suggesting that observational studies to determine associations between MDSC frequency or activity and clinical outcomes, such as response to vaccines or infection, are feasible. Cord blood MDSC are predominantly of the granulocytic type, based on CD66b and/or CD15, arginase-1 expression, and lack of CD14 expression, as well as by microscopic examination of purified cord blood MDSC, which demonstrated the appearance of neutrophils at various stages of maturation (75–77). Furthermore, granulocytic MDSC in cord blood were shown to potently suppress both T cell and NK cell responses *in vitro*, using depletion and add-back experiments. We and Rieber et al. also examined MDSC levels during infancy and early childhood by cross-sectional sampling of healthy pediatric subjects and found that MDSC likely decay to adult levels within the first few months of life (75–77).

**Figure 1:**
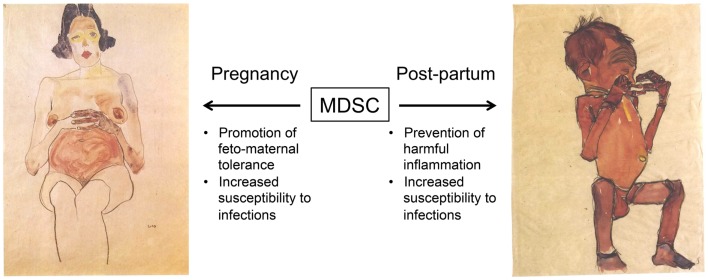
**Proposed roles of myeloid-derived suppressor cells (MDSC) during gestation and early post-natal life**. We hypothesize that an increased frequency of MDSC promotes feto-maternal tolerance during gestation. Very little is known about the expansion of maternal or fetal MDSC, or how this may be affected by the degree of HLA discordance or other factors. High levels of MDSC persist in the infant at birth, and may suppress harmful inflammation due to microbial colonization and exposure to environmental antigens. However, this may also impair the generation of protective immune responses against infections. Images by Egon Schiele (1890 – 1918), obtained through Wikimedia Commons (http://commons.wikimedia.org). Left, Red nude, pregnant (Roter akt, schwanger) 1910, private collection. Right, Newborn hiding its face with its hands (Neugeborenes die hände vor das gesicht haltend) 1910, Leopold Museum, Vienna.

These findings raise the possibility that MDSC are at least one of the mechanisms by which feto-maternal tolerance is maintained and that may underlie why neonates have impaired T cell and NK cell immunity (Figure 1). Although the possibility that MDSC suppress infant immune responses *in vivo* is highly speculative at this time, it is notable that the neonatal immune environment may be particularly prone to support the generation of MDSC. Multiple factors implicated in the expansion or activation of MDSC, including IL-10, IL-6, and TGF-β, are all increased in neonates (65, 78, 79) or fetal tissue (73). In addition, MDSC have been demonstrated to promote the development of Tregs (44–46), which are highly prevalent in the fetus and have documented importance in feto-maternal tolerance (73, 80–82). On the maternal side, higher rates of metastasis during gestation in a mouse model of melanoma was attributed to an accumulation of MDSC and their inhibition of NK cell activity (83). The potential importance of maternal MDSC in the mouse is also highlighted by studies indicating that progesterone increases MDSC (84) and that Tim-3 blockade experiments that result in fetal rejection lead to MDSC expansion (85). Finally, preliminary studies in humans have found high frequencies of MDSC in the placenta and in peripheral blood of pregnant women compared to non-pregnant controls (86, 87).

## Conclusion and Suggestions for Future Research

The specific limitations of the neonatal immune response have been implicated in the high rate of morbidity from infections in newborns and young infants. Thus, in order to reduce the enormous global burden of infant mortality due to infection, it is critical to define the mechanisms behind reduced neonatal immunity, and to identify new ways of enhancing protective immune responses in early life. Undeniably, any interventions along these lines must be approached with extreme caution in order to ensure the safety of this vulnerable population. MDSC are unambiguously important in suppressing immune responses in a variety of pathological conditions. As detailed above, it appears possible that MDSC also contribute to feto-maternal tolerance. Furthermore, MDSC may modulate early-life immune responses. We speculate that MDSC may be beneficial post-natally for preventing inflammation during colonization and microbiome establishment. However, this dampened immunity may be detrimental for mounting protective responses to vaccination and pathogenic infection. The potential of MDSC to modulate immunity in premature or young infants is particularly intriguing given the availability of drugs – e.g., retinoids, vitamin D3, sildenafil – that might be able to counteract the suppressive effects of MDSC. Indeed, if MDSC suppress protective immune responses in early infancy, it is conceivable that interventions already in wide use, e.g., vitamin A supplementation (88, 89) might affect infant health through effects on MDSC. It is also interesting to speculate that MDSC contribute to the decreased risk of graft-vs.-host disease in stem cell transplant recipients of cord blood compared to bone marrow grafts (90) or might have other therapeutic uses.

Much additional study is needed to determine whether MDSC are important in immune ontogeny, and if they might be useful targets for therapies to reduce infectious mortality in infants. Longitudinal studies of premature and term neonates are required to define the natural history and, imperatively, the physiological relevance of MDSC during early life. These cohort studies should assess not only the frequency of MDSC at different ages, but measure clinically important outcomes, such as vaccine responses and/or infectious outcomes. Importantly, such work should be performed in populations with high rates of infection and infant mortality, to ensure generalizability, and public health relevance. These studies are made more challenging by the fact that MDSC are cryosensitive, which currently necessitates testing fresh blood (91). Mouse or other animal models may also be especially useful to help evaluate the potential importance and mechanism of MDSC immune suppression during gestation and early post-natal life. Though much is known about the mechanisms of action of MDSC in cancer and other pathologic states, many questions remain, and it is unknown whether these same mechanisms can be extrapolated to MDSC functions during fetal or early post-natal life. Work is also needed to better understand the potential relationship between MDSC and neonatal APC responses, and whether MDSC interact with other suppressor cell populations of importance in immune ontogeny (Tregs, CD71^+^ erythroid cells, etc.).

In summary, recent studies suggest that MDSC are prevalent and may suppress immune responses in early life. If so, MDSC could represent one important part of the complex process of immune ontogeny and feto-maternal tolerance. In addition to their fundamental biology, these cells are of particular interest because their function can be modulated with several drugs that are widely used, whose effects on MDSC are already being evaluated in other clinical contexts. As such, future research in this field holds substantial promise.

## Conflict of Interest Statement

The authors declare that the research was conducted in the absence of any commercial or financial relationships that could be construed as a potential conflict of interest.
